# Supplementary material for: Deletion of wfs1 Impairs Oligodendrocyte Precursor Cells Dorsal Distribution and Myelination Through the wfs1-hmgcs1 Axis in Zebrafish
Source: Biology (Basel). 2026 Aug 16;15(16):1402. doi: 10.3390/biology15161402 (PMC13510151; doi:10.3390/biology15161402)
Supplement: Supplementary file 1 [file biology-15-01402-s001.zip › File S1-Transcriptome sequencing data and differentially expressed genes (DEGs) in wfs1-- zebrafish at 5 dpf/2.KEGG/wfs1KOvsWT/ALL/src/dre00480.html]

dre00480


- dre:555317

- Up regulated genes

555317(5.99248136017756)

- dre:570579

- Up regulated genes

570579\_1(5.01448411924817)

- dre:100006589

- Up regulated genes

100006589\_1(1.74277099104316)

- dre:450084

- Up regulated genes

450084\_1(4.00880053586154)

- dre:555317

- Up regulated genes

555317(5.99248136017756)

- dre:393493

- Up regulated genes

393493(3.27531260049226)
- dre:406703

- Down regulated genes

406703(-2.80714548639507)
- dre:431762

- Up regulated genes

431762\_1(2.66678410247525)

- dre:553575

- Down regulated genes

553575\_1(-3.30348159865682)

- dre:406762

- Up regulated genes

406762\_1(10.4018062903395)

- dre:100330864

- Up regulated genes

100330864(6.66489299727258)

- dre:100330864

- Up regulated genes

100330864(6.66489299727258)

Close
